# Inflammasome inhibition protects dopaminergic neurons from α-synuclein pathology in a model of progressive Parkinson’s disease

**DOI:** 10.1186/s12974-023-02759-0

**Published:** 2023-03-21

**Authors:** Alexander Grotemeyer, Judith F. Fischer, James B. Koprich, Jonathan M. Brotchie, Robert Blum, Jens Volkmann, Chi Wang Ip

**Affiliations:** 1 Department of Neurology, University Hospital of Würzburg, Josef-Schneider-Straße 11, 97080 Würzburg, Germany; 2 Krembil Research Institute, Toronto Western Hospital, University Health Network, Toronto, ON Canada; 3 Atuka Inc., Toronto, ON Canada

**Keywords:** Neurodegeneration, Movement disorder, Neuroinflammation, Parkinson’s disease, Inflammasome, Dopaminergic cells, NLRP3, MCC950, Microglia, T cells

## Abstract

Neuroinflammation has been suggested as a pathogenetic mechanism contributing to Parkinson’s disease (PD). However, anti-inflammatory treatment strategies have not yet been established as a therapeutic option for PD patients. We have used a human α-synuclein mouse model of progressive PD to examine the anti-inflammatory and neuroprotective effects of inflammasome inhibition on dopaminergic (DA) neurons in the substantia nigra (SN). As the NLRP3 (NOD-, LRR- and pyrin domain-containing 3)-inflammasome is a core interface for both adaptive and innate inflammation and is also highly druggable, we investigated the implications of its inhibition. Repeat administration of MCC950, an inhibitor of NLRP3, in a PD model with ongoing pathology reduced CD4^+^ and CD8^+^ T cell infiltration into the SN. Furthermore, the anti-inflammasome treatment mitigated microglial activation and modified the aggregation of α-synuclein protein in DA neurons. MCC950-treated mice showed significantly less neurodegeneration of DA neurons and a reduction in PD-related motor behavior. In summary, early inflammasome inhibition can reduce neuroinflammation and prevent DA cell death in an α-synuclein mouse model for progressive PD.

**Graphical Abstract:**

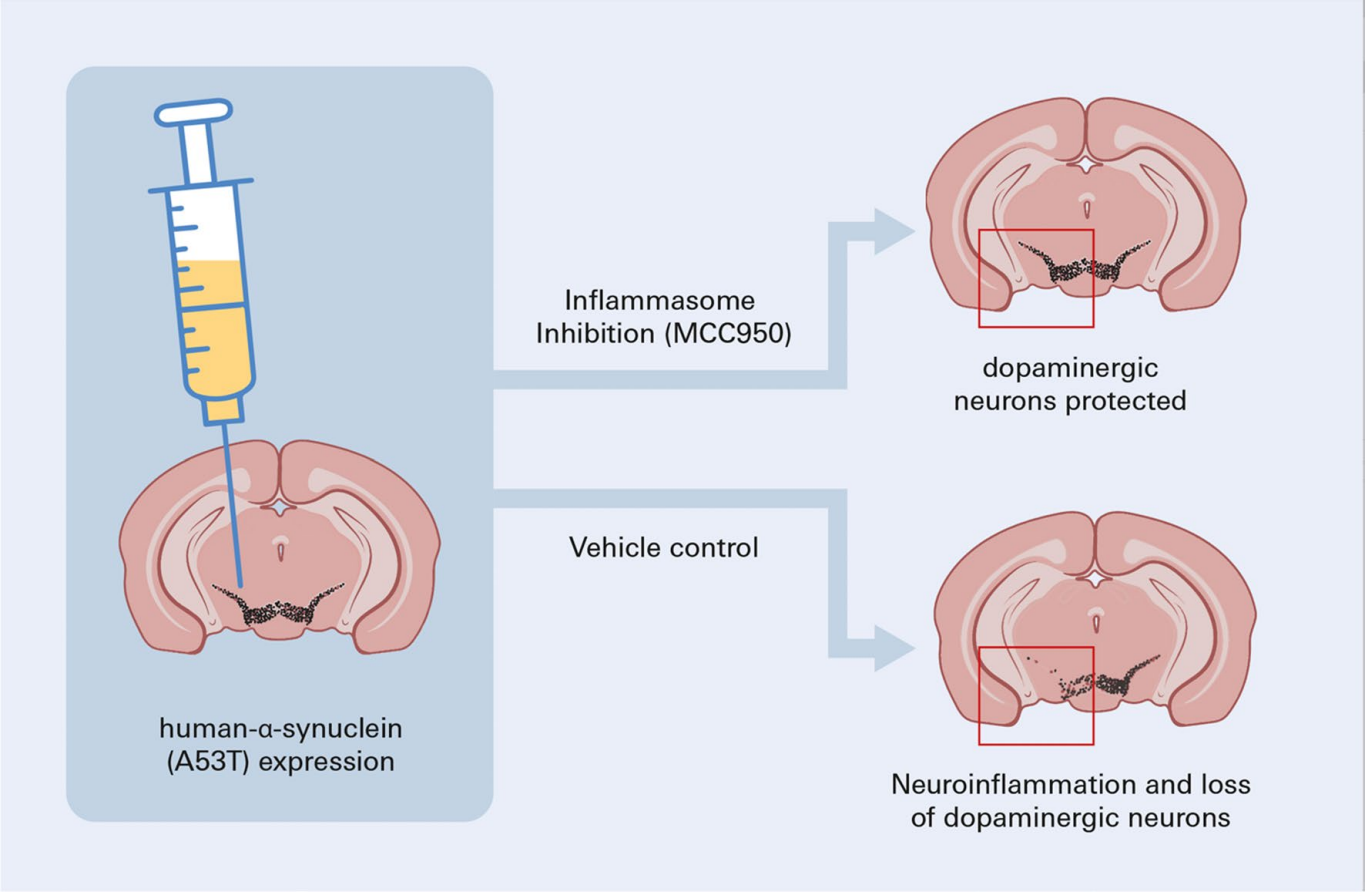

## Introduction

Parkinson’s disease (PD) is the most common neurodegenerative movement disorder. Dopaminergic (DA) cell death in the substantia nigra (SN) is widely accepted to be closely related to aggregation of pathological α-synuclein (p_ath_αSYN) and neuroinflammation [1, 2]. Furthermore, antecedent and concomitant inflammation have been shown to contribute to neurodegeneration in PD [3–9]. An important cellular sensor for inflammatory events is the NLRP3 (NOD-, LRR- and pyrin domain-containing 3)-inflammasome, which serves as a cellular innate immune component and is upregulated in a proinflammatory environment [10, 11]. Recently, NLRP3 upregulation in microglia was shown to induce strong upregulation of inflammatory cytokines, transcription factors, and critical members of the inflammasome pathway, and that the inflammasome contributes to DA cell loss in the toxin-based MPTP (1-methyl-4-phenyl-1,2,3,6-tetrahydropyridine) PD mouse model [12]. The herein used diarylsulfonylurea-containing NLRP3-inhibitor MCC950 has been already characterized earlier [13, 14]. It directly blocks the ATPase domain of NLRP3, inhibiting (non-)canonical activation of the NLRP3 inflammasome [15, 16]. However off-target activity—non-competitive inhibition of carbonic anhydrase 2—has been found in an in vitro study [17]. MCC950 was found to attenuate DA cell loss in the MPTP mouse model, highlighting the role of the NLRP3-inflammasome [18]. It has also been shown that the NLRP3 pathway and microglia are triggered in vivo due to fibrillar α-SYN and that NLRP3 inhibition, by MCC950, reduces inflammation and aggregation of fibrillar α-SYN [19]—as a prophylactically initiated treatment. However, innate and adaptive immune response together and specifically the effect of T cells in relation to αSYN aggregation in a progressive PD model has yet to be examined.

To investigate how p_ath_αSYN modulates immune function and thereby affects the function of DA neurons in the SN, we implemented a viral vector-based progressive PD model expressing the pathological human A53T-αSYN (hαSYN) [20, 21]. Our recent in vivo and in vitro research indicated that p_ath_αSYN-specific T cell responses cause DA neurodegeneration in the SN, thereby contributing to PD-like pathology [6]. Here, we have evaluated systemic inflammasome inhibition by MCC950 to understand the in vivo relationship between p_ath_αSYN and neuroinflammation—especially as it is known that NLRP3 is required for the functionality of T cells [22]. Furthermore, CD8^+^ T cells are involved in the early stages of PD as they are found to precede p_ath_αSYN in early PD patients [4].

## Materials and methods

### Ethics statement

All experiments were performed in accordance with the guidelines of the European Union and approved by our institutional Animal Care, the Utilization Committee and the Regierung von Unterfranken, Würzburg, Germany (License number: 55.2DMS-2532-2-221). All experiments were conducted in the ‘Zentrum für Experimentelle Molekulare Medizin’ Würzburg, Germany.

### Animals

**Fig. 1:**
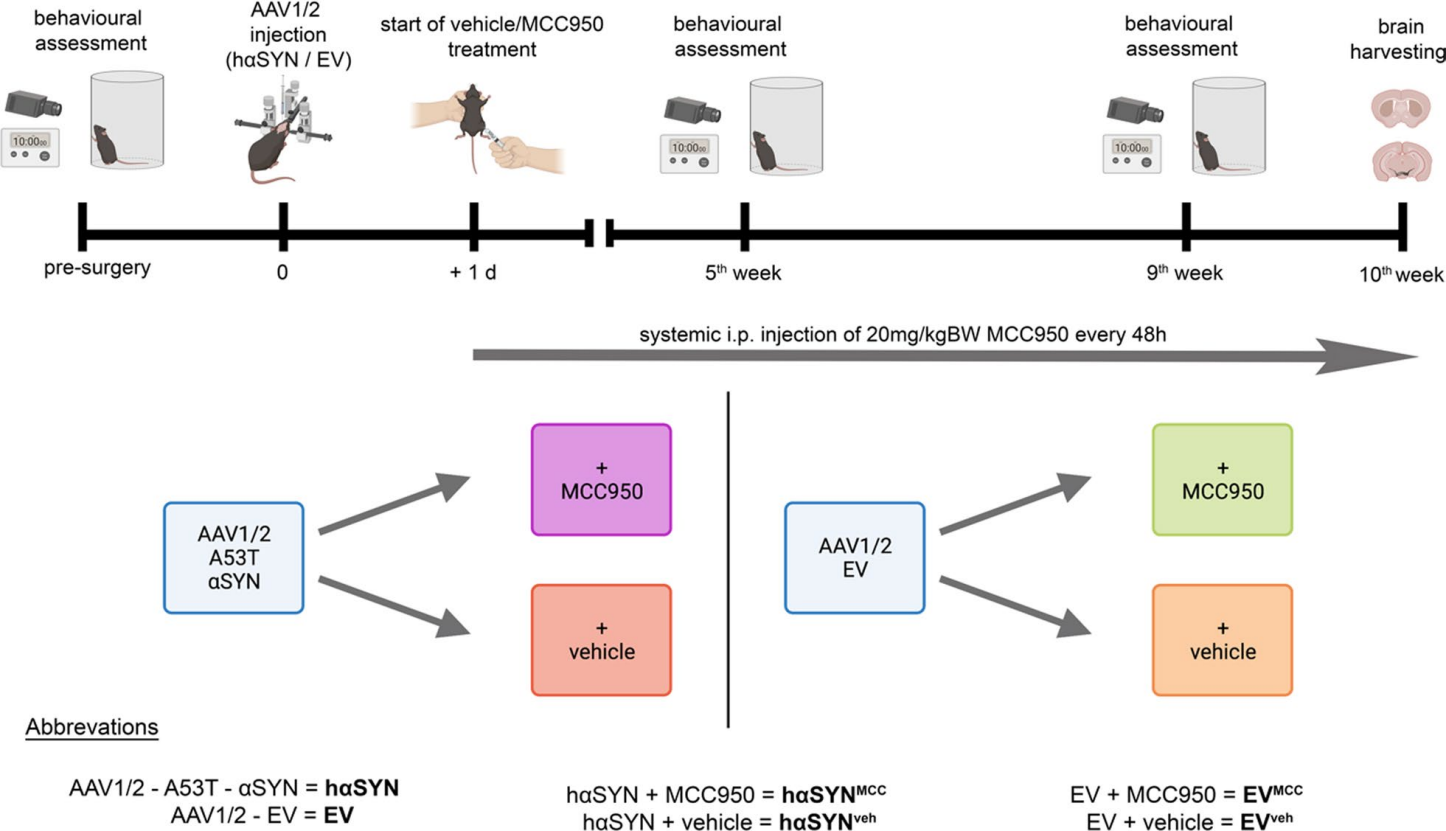
Overview of the experimental protocol. The upper section illustrates the temporal experimental setup with the experimental foci. Systemic intraperitoneal injection of MCC950 started 24 h after AAV1/2 viral vector injection in the right substantia nigra. The lower section illustrates the experimental grouping. Created with BioRender.com

Healthy male C57Bl/6J mice (Charles River, Sulzfeld, Germany) were kept in a near pathogen-free environment under standard conditions (21 °C, 12 h/12 h light–dark cycle) and provided chow and water ad libitum. A viral vector injection was administered in 11–12-week-old mice. To avoid possible hormonally induced bias regarding behavioral testing and the investigation of neurodegeneration only male animals were used. Mice were euthanized and investigated 10 weeks after viral vector injection (Fig. 1). To investigate whether the NLRP3-mediated inflammasome contributes to synuclein pathology in the AAV1/2-A53T-αSYN model, wild-type C57/Bl6-mice were split into four experimental groups (Fig. 1). In one group, mice expressing the A53T mutant of human α-synuclein in the SN (hαSYN mice) modelled the diseased condition, expressing pathology with a PD-like phenotype. In the experimental group, hαSYN mice were systemically treated for 10 weeks with the NLRP3-inhibitor MCC950 (hαSYN^MCC^) (Fig. 1). Vehicle-treated hαSYN mice (hαSYN^veh^) served as disease control. Groups treated with empty AAV1/2-vector (EV), with or without MCC950 treatment, served as model (EV^MCC^) or treatment control (EV^veh^) (Fig. 1).

### Viral vector injection and drug delivery

We used AAV1/2 vectors as described by Koprich et al. [20] (custom order from GeneDetect Ltd [Auckland, New Zealand]). Viral vector injections were performed as previously described [21]. Mice, deeply anaesthetized with isoflurane, were placed on a stereotactic frame (Neurostar^®^). The AAV1/2 vectors were injected unilaterally into the right SN using an injector-controlled 5 µl Hamilton syringe (Merck; cat. #26200-U). The injection rate was 0.25 µl/min with either 2 µl of AAV1/2 (empty vector, EV) or AAV1/2 carrying human A53T-αSYN (hαSYN), each with 5.16 × 10^12^ genome copies (gc) per ml. Coordinates were chosen according to Paxinos and Franklin [23]. Coordinates (mm) were: anterior–posterior -3.1; medio-lateral 1.4; dorso-ventral (DV) 4.4. In total, 43 mice were injected with hαSYN and 43 mice with EV. Since the half-life of MCC950 is relatively short (3.27 h) and oral bioavailability is of about 68% only, we here chose to administer MCC950 i.p. every second day as described [13]. From day 1 post-virus injection, MCC950 (20 mg/kg body weight, SigmaAldrich, cat. #256373-96-3) was freshly dissolved in 0.9% sterile NaCl and injected intraperitoneal every other day (Fig. 1). An equal amount of 0.9% NaCl was used as vehicle control (Fig. 1). Analgesia was performed by injecting carprofen (5 mg/kg bodyweight) subcutaneously.

### Behavioral analysis

#### Cylinder test

Spontaneous forepaw use was assessed using the cylinder test before and 9 weeks after the EV or hαSYN injection, as previously described [6, 21] (Fig. 1). Data were collected from two temporally separated and independent cohorts. Each cohort included all experimental *groups.*

#### Accelerating rotarod

Mice were subjected to the rotarod performance test before and 9 weeks after the EV or hαSYN injection. For this, mice were trained for 2 days prior to pre-operative measurements. Pre-operative measurements were recorded on the third day after the 2-day training session. Five consecutive runs were conducted on each training or testing day. Mice were placed on a rotarod with an accelerating speed ranging from 5 rounds per minute (rpm) to 50 rpm for the duration of 300 s (RotaRod Advanced, TSE-systems) and latencies to fall were automatically recorded. Data are shown as latencies of 9th week relative to the pre-operative measurement for each mouse.

### Tissue preparation and immunohistochemistry

Mice were sacrificed at the age of 21–22 weeks, 10 weeks after viral vector injection. After transcardial perfusion with 0.1 M phosphate-buffered saline (PBS) containing 0.34%v/v heparin (Heparin-Sodium-25000, Ratiopharm), brains were dissected in a frontal (striatal) and dorsal part at Bregma − 1.06 mm. The dorsal part containing the SN was post-fixed in 4% paraformaldehyde (PFA), buffered with 0.1 M PBS for 2 days, and then rinsed in 30% sucrose/PBS for another 2 days at 4 °C. After embedding in Tissue-Tek^®^ (O.C.T Compound Sakura), the dorsal part (Bregma − 2.06 mm to − 4.04 mm) was serially cut into 40 μm-thick coronal sections on a cryostat (Leica 3050, Leica Biosystems). For tyrosine hydroxylase (TH) and Nissl double staining, the sections of one series (out of five) was used. After three washing steps with 0.1 M PBS, sections were treated with blocking solution (10% normal goat serum (NGS), 2% bovine serum albumin (BSA), 0.5% Triton X-100 (SigmaAldrich, cat. #X100) in 0.1 M PBS (PBT) for 1 h. Primary antibody (rabbit anti-tyrosine hydroxylase; Abcam, cat. #ab112; RRID:AB_297840, 1:1000) was incubated in 2% NGS, 2% BSA in PBT, overnight at room temperature (RT). As a secondary antibody, biotinylated goat‐anti‐rabbit (Vector Laboratories, cat. #BA-1000; RRID:AB_2313606) was used at 1:100 dilution in 2% NGS, 2% BSA in PBT, for 2 h at RT. After incubation for 2 h in an avidin/biotin solution (Standard Ultra-Sensitive ABC Staining Kit (32050), Thermo Fisher Scientific), the sections were developed with diaminobenzidine (DAB)‐HCl and H_2_O_2_ (Peroxidase Kit, Vector Laboratories, cat. #SK-4100). To complete the Nissl staining, sections were incubated in cresyl violet solution (1 g of cresyl violet + 10 ml of 100% acetic acid and 1 L of distilled water; Certistain^®^, SigmaAldrich, cat. #105235) for 30 min at RT.

For immunohistochemical staining of brain tissue for CD4^+^, CD8^+^, CD11b^+^ cells, and TH^+^ striatal fibers, brains were snap frozen. The striatum and SN were serially cut into 10 µm-thick coronal sections. Stainings of the striatum were performed at + 0.14 mm from Bregma according to Paxinos and Franklin [23]. After fixation with either acetone (t cell labeling) or 4% PFA (labeling of myeloid cells and TH fibers) for 15 min and blocking (TH fibers [2% BSA and 10% NGS], CD4/8 [5% BSA], CD11b [1% BSA, 5% NGS]), sections were incubated overnight with rat anti-mouse CD4 (1:1000, Bio-Rad, cat. #MCA1767; RRID:AB_322769), rat anti-mouse CD8 (1:500, Bio-Rad, cat. #MCA609G; RRID:AB_321407), rat anti-mouse CD11b (1:100, Bio-Rad, cat. #MCA711; RRID:AB_321292), or rabbit anti-TH antibodies. As secondaries, biotinylated rabbit anti-rat or goat anti-rabbit antibodies were used (Vector Laboratories, cat. #BA-4001; RID:AB_10015300 and BA-1000; RRID:AB_2313606), respectively. DAB staining was performed as described above. Alternative DAB Substrate Kit (abcam, cat. #ab64238) and ABC Kit (Vectastain Elite ABC-HRP Kit, Peroxidase; Vector Laboratories; cat. #VEC-PK-6100) was used in case of CD11b staining.

### Immunofluorescence stainings

Immunofluorescence (IF) stainings of TH, CD11b, and DAPI (4′,6-diamidino-2-phenylindole) on fresh-frozen sections were performed as described before [6, 21]. In brief, 10 µm serial-cut sections of the SN were post-fixed with 4% PFA/PBS and blocked with 10% NGS and 2% BSA for 1 h at RT. Primary antibodies (chicken anti-mouse TH; abcam cat. #76442; RRID:AB_1524535, 1:500; rat anti-mouse CD11b) were incubated in a humidified chamber overnight at 4 °C. Secondary antibodies (anti-chicken Alexa 488 (Invitrogen, cat #A11039; RRID:AB_253409) and goat anti-rat Alexa 647 (abcam cat. #150167; RRID:AB_286429); 1:300 each) were used for 1 h at RT.

For NLRP3 staining, the labeling protocol was as follows: after 5 min acetone fixation, 10 µm fresh-frozen coronal cryo-sections were blocked with 5% BSA in PBS containing 0.2% Triton X-100, as previously described [24]. In brief, mouse anti-NLRP3/NALP3 (1:100, AdipoGen, cat. #AG-20B-0014-C; RRID:AB_2885199), rat anti-mouse CD11b (1:300, Bio-Rad, cat. #MCA74G; RRID:AB_321293), rat anti-mouse CD8 (1:500, Bio-Rad, cat. #MCA609G; RRID:AB_321407), or rat anti-mouse CD4 were incubated in 1% BSA/PBS for 1 h. Goat anti-mouse Alexa 488 (1:100, Jackson ImmunoResearch Labs, cat. #115-545-166; RRID:AB_2338852) in 1% BSA/PBS and goat anti-rat Alexa 647 (1:300) were incubated for 1 h. Cell nuclei were labeled with DAPI (SigmaAldrich, cat. #D8417) for 10 min at RT.

Staining against αSYN, TH, microtubule-associated protein 2 (MAP2) and DAPI was performed for confocal imaging. 40 µm post-PFA-fixed interval section (1/5) were used. Sections were incubated overnight at 4 °C with chicken anti-mouse TH (1:500), rabbit anti-human αSYN (1:10,000, SigmaAldrich cat. #S3062, RRID:AB_477506) and mouse anti-mouse MAP2 (SigmaAldrich, clone AP20, #M1406, RRID:AB_477171, 1:200). Subsequently, the tissue was incubated with the following secondary antibodies for 2 h at RT: anti-chicken Alexa 488 (1:300), goat anti-rabbit Cy3 (1:300, Jackson ImmunoResearch, #111-165-144, RRID:AB_2338006) and donkey anti-mouse Cy5 (1:300, Jackson ImmunoResearch #715-175-150, RRID:AB_2340819). Cell nuclei were labeled with DAPI (SigmaAldrich #D8417) for 10 min at RT.

### Confocal microscopy

For confocal image acquisition, an inverted IX81 microscope equipped with an Olympus FV1000 confocal laser scanning system, a FVD10 SPD spectral detector, and 405, 473, 559, and 635 nm diode lasers was used. Confocal images (x, y, z) were acquired in 12-bit with an Olympus UPLSAPO 20X objective (numerical aperture: 0.75). To improve presentation, maximum intensity projection images were adjusted in brightness and contrast and are presented as RGB images (8-bit per color channel).

### Proteinase K digestion and imaging

For proteinase K (PK) digestion and imaging, 10 µm fresh-frozen sections on object slides were first dried for 2 min at RT. Afterwards, sections were fixed with 4% freshly prepared PFA for 20 min at RT. After three washing steps for 5 min each with 1 × PBS, sections were incubated with PK solution (20 µg/ml in 1 × PBS; SigmaAldrich cat. #P2308) for 10 min in a humidified chamber at 37 °C. One section, out of four per object slide, was spared from PK solution to remain intact. PK solution was removed in three washing steps (5 min each) with ice-cold 1 × PBS. Subsequently for immunofluorescence staining, sections were incubated overnight at 4 °C with chicken anti-mouse TH (1:500) and rabbit anti-human αSYN (1:5,000). Afterwards, the tissue was incubated with secondary antibodies (anti-chicken and goat anti-rabbit Cy3 (see above) 1:300) for 1 h at RT.

PK-digested IF-stained sections were acquired in 16-bit with an Axio Imager.M2 (20X, air) system. For each animal analyzed, one untreated 10 µm brain section was captured to define the region of SN and to compare αSYN intensity before and after digestion within the same region of one PK-treated slice from the same object slide.

### Stereological quantification of TH^+^ neurons

For analysis of TH^+^ neurons in the SN pars compacta (SNpc), a Stereo Investigator software package (version 11.07; MicroBrightField Biosciences, Williston, VT, USA) was used. For counting of DA neurons, the investigator was blinded to the individual animals and groups. The hemisphere ipsilateral to the viral vector injection was analyzed. 40 µm sections in 200 µm intervals, representing the entire SN, were used for cell counting. Sections were analyzed with a 100X/1.25 numerical aperture objective on a BX53 microscope (Olympus). The counting parameters were: grid size 110 × 110 μm; counting frame 50 × 50 μm; guard zone 2 μm zone. A Gundersen coefficient of error (for m = 1) of < 0.1 was accepted.

### Analysis of immunohistochemical stainings

CD4^+^ and CD8^+^ T cells were quantified using a BH2 light microscope (Olympus) and a 64X objective. The area of the structure was calculated using NIH ImageJ (“Fiji” version 2.0-rc-69/1.52p, RRID:SCR_002285). CD11b^+^ cells of the SN were manually counted using a 40X objective and a counting grid. The optical density (OD) of the DA terminals in the striatum was determined as previously described [21]. The OD of the corpus callosum was used as background. Representative images were acquired with a Glissando whole-slide scanner (40X, Objective Imaging).

### Analysis of immunofluorescence stainings

For all procedures described below, images were acquired in 16-bit with an Axio Imager.M2 (20X, air, epifluorescence) and split into different channels if not stated otherwise. Maximum intensity images were merged and background corrected, and NIH ImageJ was used for analysis.

For analysis of cellular properties of CD11b^+^ cells, the SNpc was selected with the polygon tool using TH^+^ cells as an indicator. The region of interest (ROI) containing the SNpc was transferred to the CD11b^+^ image and merged with the DAPI label. Subsequently, the SNpc area was analyzed and all CD11b^+^ cells with a definite nuclear DAPI signal were analyzed. The CD11b^+^ perikaryon area and corresponding DAPI^+^ nucleus area were both defined using the NIH ImageJ polygon tool and an ROI list was created. The subsequent analysis was performed by calculating the proportion of the perikaryon area after subtraction of the DAPI^+^ nuclear area [(Perikaryon area − Nucleus area)/Perikaryon area = proportion of Perikaryon relative to nucleus area]. This proportion was used to compare the relative change in the perikaryon between the groups.

For analysis of NLRP3^+^ cells, an ROI square of 0.2 mm^2^ on the lateral region of the SN was selected in the same region for all images. NLRP3^+^ cells, identified as DAPI nuclei with surrounding cellular NLRP3^+^ signals, were counted manually with the counting tool.

For analysis of αSYN intensity, SNpc ROIs were aligned along the TH^+^ cell signal captured from the undigested section for each animal individually. This ROI was then used to analyze the mean αSYN intensity before digestion and transferred to an image of a digested section to analyze the mean αSYN intensity after digestion. Therefore, the mean intensity of αSYN immunoreactivity labels was captured in both cases to evaluate the overall αSYN immunoreactivity per SNpc.

Analysis of immunoreactivity intensity and absolute cell number of αSYN and TH^+^ cells was performed on confocal stack images (as described earlier) of (undigested) 40 µm interval sections. Images were split into different channels. Maximum intensity images were merged and background corrected. SNpc was defined along the TH^+^ cell signal. Afterwards, each cell within this SNpc-ROI was selected and defined using the polygon tool, creating individual cell-ROIs. Absolute number of cell-ROIs were counted and the integrated density of the immunolabels was calculated for each cell to evaluate the immunoreactivity of αSYN on a cellular level. This cell-ROI data set was then transferred onto the maximum intensity images of MAP2 for analysis of cellular co-expression, followed by TH^+^ cell analysis.

### Statistics

For statistical analyses, Graph Pad Prism Version 9.1.2 was used. Normality was determined by the Shapiro–Wilk test. Normal distributed data sets were statistically analyzed using one-way ANOVA and the Tukey’s multiple comparison test. Non-parametric data was analyzed using the Kruskal–Wallis test followed by Dunn’s post-test. The graphs indicate mean ± standard error of the mean (SEM). (*) *P* < 0.05, (**) *P* < 0.01, (***) *P* < 0.001, and (****) *P* < 0.0001 were considered significant p values.

## Results

### Systemic MCC950 treatment reduces NLRP3 and CD11b^+^ microglia activation in the SN of hαSYN mice

**Fig. 2:**
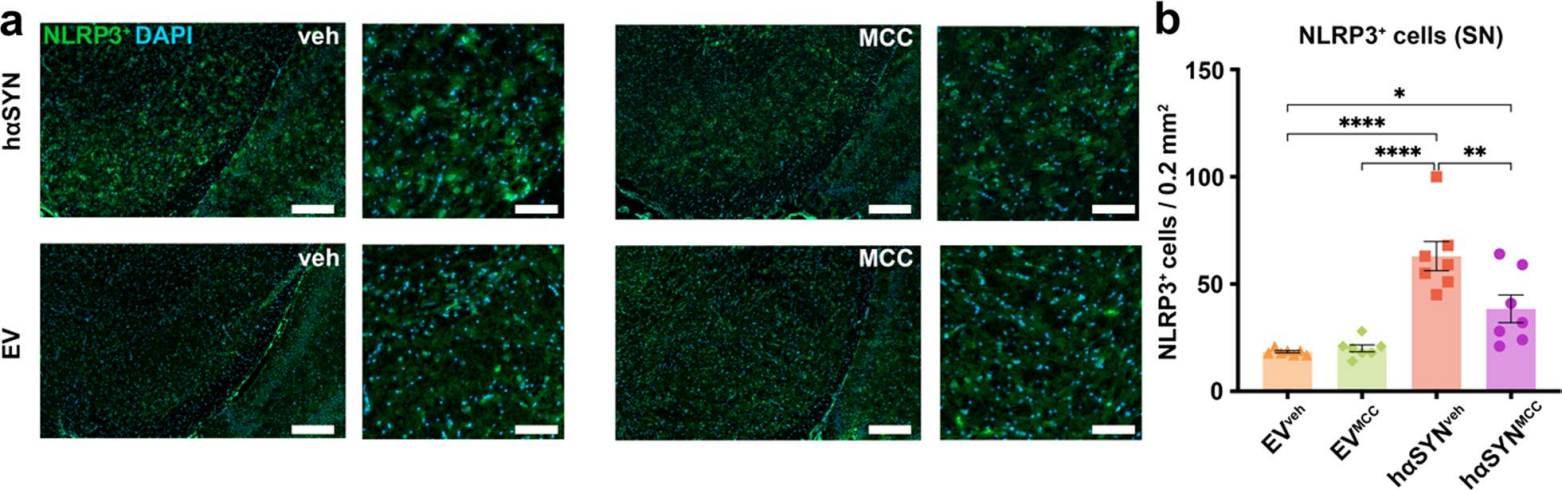
MCC950 reduces the number of NLRP3-positive cells in the substantia nigra (SN) of hαSYN mice. **a** Representative images of SN brain sections stained with NLRP3. **b** Number of NLRP3^+^ cells (per area) in the SN of all experimental groups are indicated. Asterisks (*) indicate significance level. Statistical analysis by one-way ANOVA followed by Tukey’s multiple comparisons test: *F*(3, 24) = 19.04, *P* < 0.0001; *P < 0.05, **P < 0.01, ***P < 0.001, ****P < 0.0001. Data are shown as mean ± standard error of the mean. *n* = 7 biologically independent, randomly selected samples for each group investigated. Scale bars: overview (left) = 250 µm; detail (right) = 100 µm

**Fig. 3:**
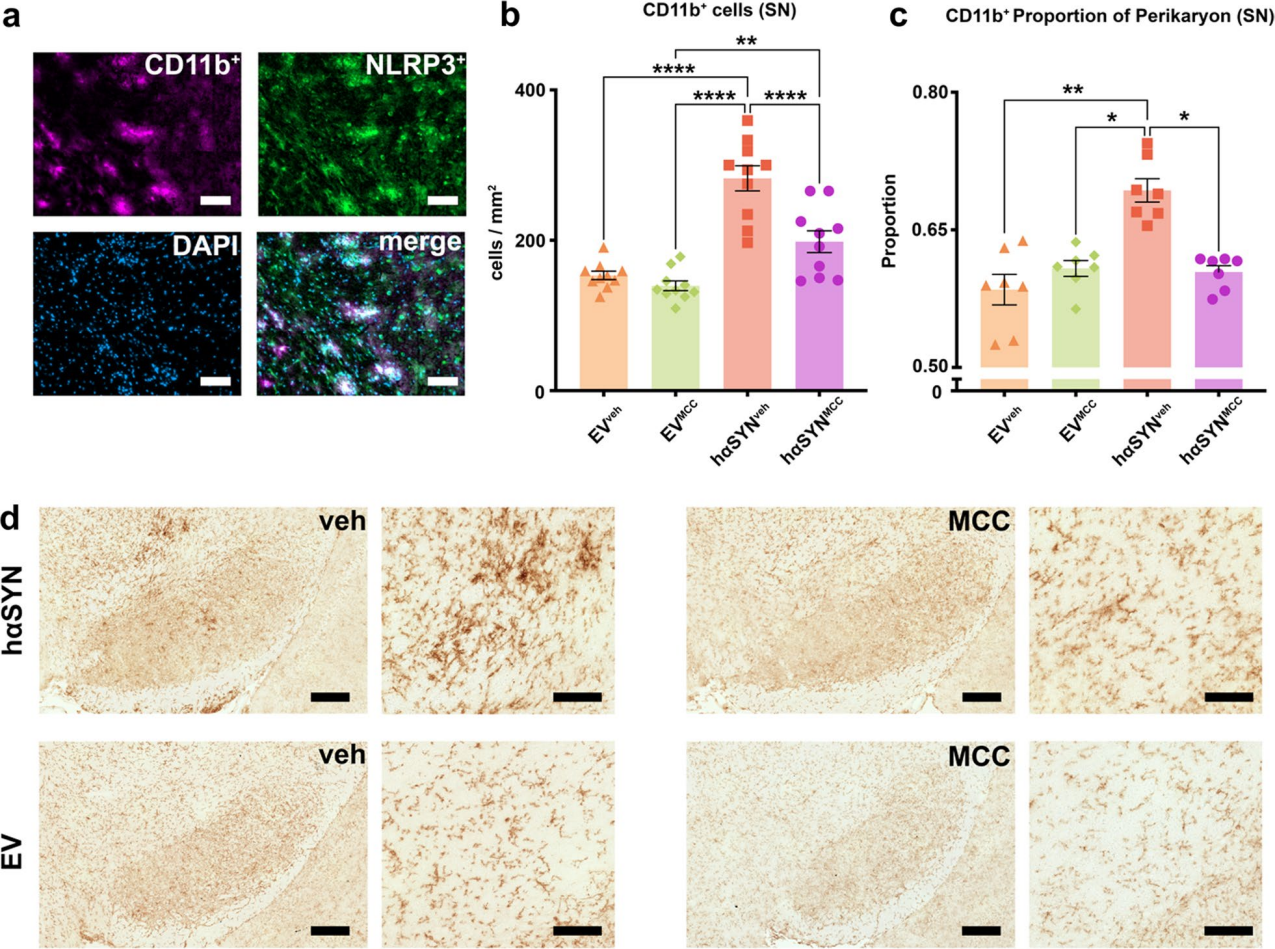
Reduced CD11b^+^ microglia activation in hαSYN mice after MCC950 treatment. **a** Immunofluorescence co-staining of NLRP3, CD11b, and DAPI in hαSYN^veh^ mice. Scale bars = 50 µm. **b** Bar graph showing quantification of CD11b^+^ microglia in the substantia nigra (SN) of the different groups (*n* = 10). Statistical analysis by one-way ANOVA followed by Tukey’s multiple comparisons test: *F*(3,36) = 29.44, *P* < 0.0001. **c** Cell size indicated by the perikaryon to nucleus proportion of CD11b^+^ microglia (*n* = 7). Statistical analysis by Kruskal–Wallis test followed by Dunn’s multiple comparisons test: Kruskal–Wallis test = 15.83, four groups, *P = *0.012. **d** immunohistochemical diaminobenzidine staining of 10 µm SN sections against CD11b. Scale bars: overview (left) = 250 µm, detail (right) = 100 µm. *P < 0.05, **P < 0.01, ***P < 0.001, ****P < 0.0001

To assess target engagement and the anti-inflammatory properties of NLRP3 inhibition, we analyzed the SN of hαSYN^MCC^ mice and controls 10 weeks after AAV injection for cellular NLRP3 expression and neuroinflammation (Fig. 2). In hαSYN^MCC^ mice, the number of observed NLRP3-positive cells was 39.0% lower than in hαSYN^veh^ mice (*P* < 0.01) (Fig. 2). This indicated that MCC950 treatment reduced NLRP3 expression and showed target engagement in hαSYN^MCC^ mice. Microglia are known to display increased NLRP3 expression in inflammatory-degenerative brain diseases such as Alzheimer’s and multiple sclerosis [25, 26]. We thus co-labeled NLRP3 and the microglia activation marker CD11b in the SN of hαSYN^veh^ mice (Fig. 3a) to verify expression of NLRP3 in microglia. Quantitative analysis of microglia revealed a reduced absolute number (29.8%, *P* < 0.0001) and cell size (~ 12.8%, *P* < 0.05) of CD11b^+^ microglia in the SN of hαSYN^MCC^ mice (Fig. 3b–e) compared to hαSYN^veh^ mice. These results indicate efficacy of MCC950 in reducing NLRP3 expression and the innate immune response.

### Inflammasome inhibition reduces the T cell response in the SN of hαSYN mice

**Fig. 4:**
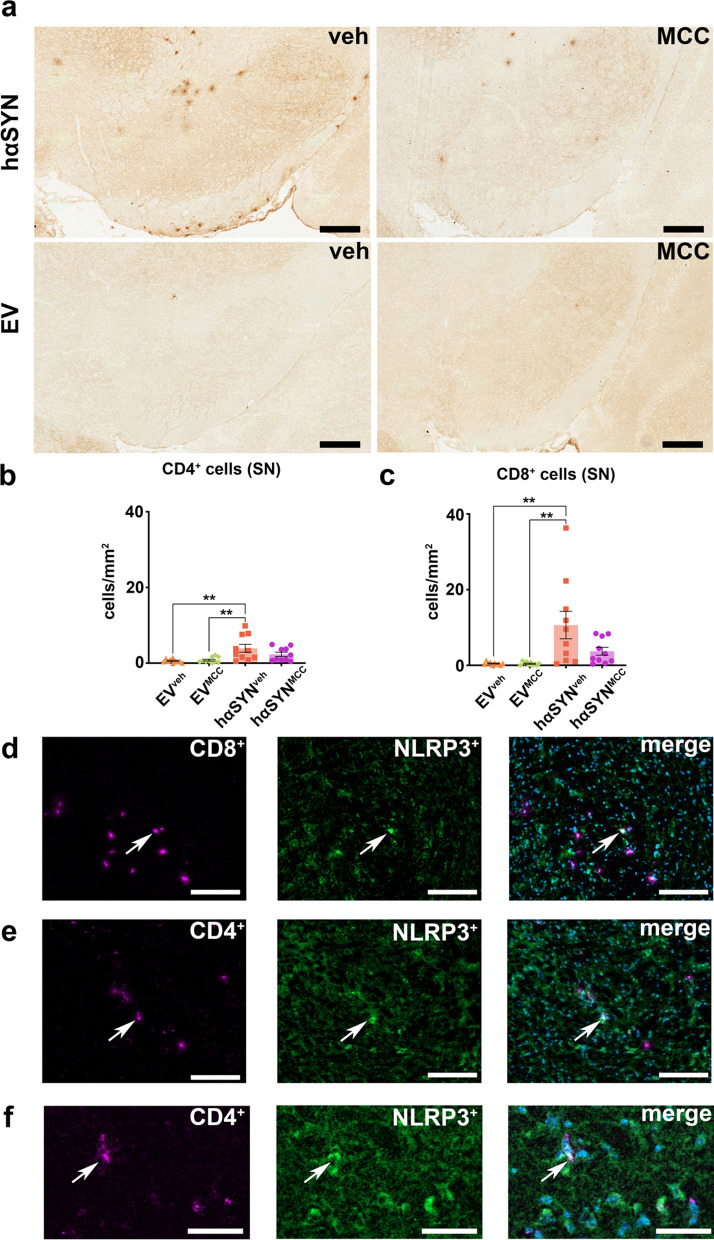
Visualization and analysis of T cell immunoreactivity hαSYN and empty vector (EV) mice. **a** Representative RGB color images of immunohistochemical staining against CD8^+^ T cells. Scale bars: 250 µm. **b** Bar plot of counting analysis of CD4^+^ T cells in the substantia nigra (SN). Statistical analysis by one-way ANOVA followed by Tukey’s multiple comparisons test: *F*(3,36) = 6.381, *P = *0.0014, *n* = 10 mice/group.** c** Bar plot of counting analysis of CD8^+^ T cells in the SN. Statistical analysis by one-way ANOVA followed by Tukey’s multiple comparisons test: *F*(3,36) = 6.487, *P = *0.0013, *n* = 10 mice/group. **d** Representative example 8-bit images of immunofluorescence (IF) co-staining against CD8 and NLRP3; Scale bars = 150 µm. **e, f** Representative example 8-bit images of IF co-staining against CD4 and NLRP3. Overview (left): Scale bars = 150 µm; detail in **f**: Scale bars: 50 µm. *P < 0.05, **P < 0.01, ***P < 0.001, ****P < 0.0001. Data are shown as mean ± standard error of the mean

In addition to test if the attenuation of the innate immune response directly related to reduced NLRP3 expression, we also observed a clear impact of MCC950 on the adaptive immune system. While hαSYN^veh^ mice demonstrated a significant increase of CD4^+^ and CD8^+^ T cell counts in the SN compared to EV^veh^ and EV^MCC^ mice (CD4^+^: *P* < 0.01, *P* < 0.01; CD8^+^: *P* < 0.01, *P* < 0.01), CD4^+^ and CD8^+^ T cell numbers in the SN of hαSYN^MCC^ mice were decreased and did not differ significantly from T cell number of EV^MCC^ and EV^veh^ mice (Fig. 4a–c). Notably, NLRP3 was expressed by some CD4^+^ and CD8^+^ cells in hαSYN^veh^ mice, as shown in Fig. 4d–f.

### Inflammasome inhibition prevents the loss of TH^+^ neurons and mitigates motor deficits in hαSYN mice

**Fig. 5:**
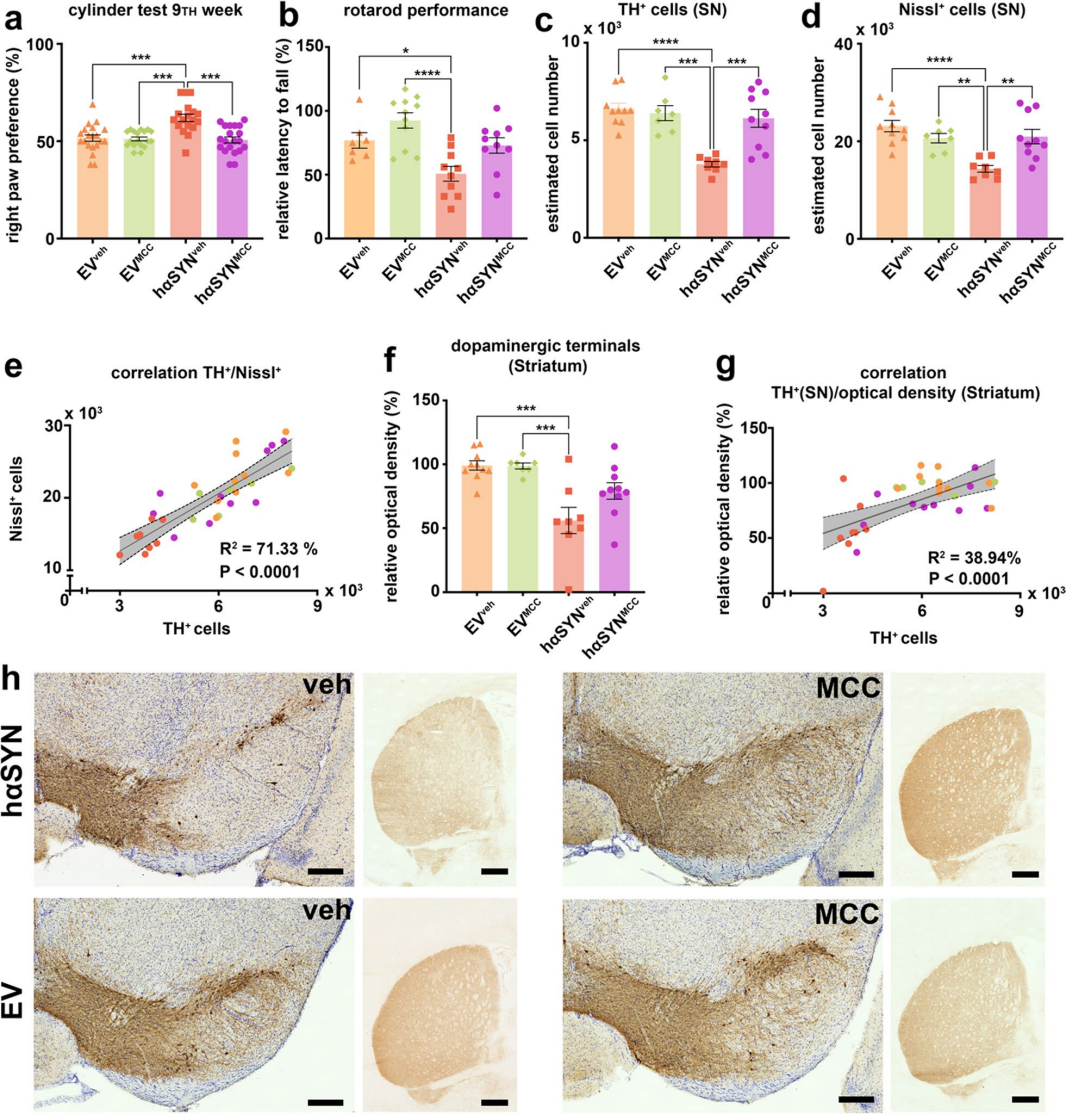
Analysis of behavior and DA neuroprotection related to NLRP3-inflammasome inhibition. **a** Evaluation of right paw preference in the cylinder test. Statistical analysis by Kruskal–Wallis test followed by Dunn’s multiple comparisons test: Kruskal–Wallis statistic = 23.30, *P* < 0.0001, n—numbers: empty vector (EV)^veh^ = 20, EV^MCC^ = 18, hαSYN^veh^ = 18, hαSYN^MCC^ = 20. **b** Evaluation of relative latency to fall from the rotarod. Statistical analysis by one-way ANOVA followed by Tukey’s multiple comparisons test: *F*(3, 34) = 8.845, *P = *0.0002, n—numbers: EV^veh^ = 7, EV^MCC^ = 11, hαSYN^veh^ = 10, hαSYN^MCC^ = 10. **c** Estimated cell count of TH^+^ cells in the substantia nigra (SN). Statistical analysis by one-way ANOVA followed by Tukey’s multiple comparisons test: *F*(3,31) = 13.33, *P* < 0.0001, n—numbers: EV^veh^ = 10, EV^MCC^ = 7, hαSYN^veh^ = 8, hαSYN^MCC^ = 10. **d** Estimated Nissl^+^ cell count. Statistical analysis by one-way ANOVA followed by Tukey’s multiple comparisons test: *F*(3,31) = 9.815, *P = *0.0001, n—numbers: EV^veh^ = 10, EV^MCC^ = 7, hαSYN^veh^ = 8, hαSYN^MCC^ = 10. **e** Correlation analysis of estimated TH^+^ and Nissl^+^ cell count. **f** Relative optical density of anti-TH immunoreactivity on the ipsilateral side compared to the contralateral side of the striatum. Statistical analysis by one-way ANOVA followed by Tukey’s multiple comparisons test: *F*(3,31) = 9.606, *P = *0.0001, n—numbers: EV^veh^ = 10, EV^MCC^ = 7, hαSYN^veh^ = 8, hαSYN^MCC^ = 10. **g** Correlation analysis of estimated TH^+^ cell count and optical density of anti-TH immunoreactivity in the corresponding striatum. **h** Representative RGB color images of right SN and striatum for each group. Scale bars: 200 µm (SN) and 500 µm (striatum). *P < 0.05, **P < 0.01, ***P < 0.001, ****P < 0.0001. Data are shown as mean ± standard error of the mean

We investigated whether MCC950 could prevent PD-like behavior in hαSYN mice and found significant reduction of motor deficits in hαSYN^MCC^ compared to hαSYN^veh^ mice (Fig. 5a). The cylinder test demonstrated a significantly increased right paw preference of 20% and the rotarod performance test showed a decreased latency to fall in hαSYN^veh^ mice compared to hαSYN^MCC^, EV^veh^ and EV^MCC^ controls (cylinder test: all *P* < 0.001; rotarod: *P = *0.0547, *P* < 0.05, *P* < 0.0001; Fig. 5a, b). Subsequently, we investigated the efficacy of MCC950 treatment in ameliorating SN neurodegeneration, as normal behavior was found be preserved in hαSYN^MCC^ mice. The total amounts of TH^+^ cells and Nissl^+^ cells in hαSYN^veh^ mice were significantly decreased, by 38.4% and 31.5% respectively, compared with hαSYN^MCC^ mice (*P* < 0.001 and *P* < 0.01; Fig. 5c, d). Furthermore, there was a significant correlation between the amount of Nissl^+^ and TH^+^ cells in an overall analysis for all groups (Fig. 5e). Relative OD (as an indicator for the density of DA terminals) in the striatum was also preserved in hαSYN^MCC^ mice, although not significantly compared to hαSYN^veh^ (*P = *0.0677, Fig. 5f). Consistent with this finding, there was a weaker but still significant correlation between TH^+^ cell number and relative OD (Fig. 5g). These data indicated a neuroprotective role of MCC950 in hαSYN^MCC^ mice.

### Inflammasome inhibition attenuates αSYN aggregation

**Fig. 6:**
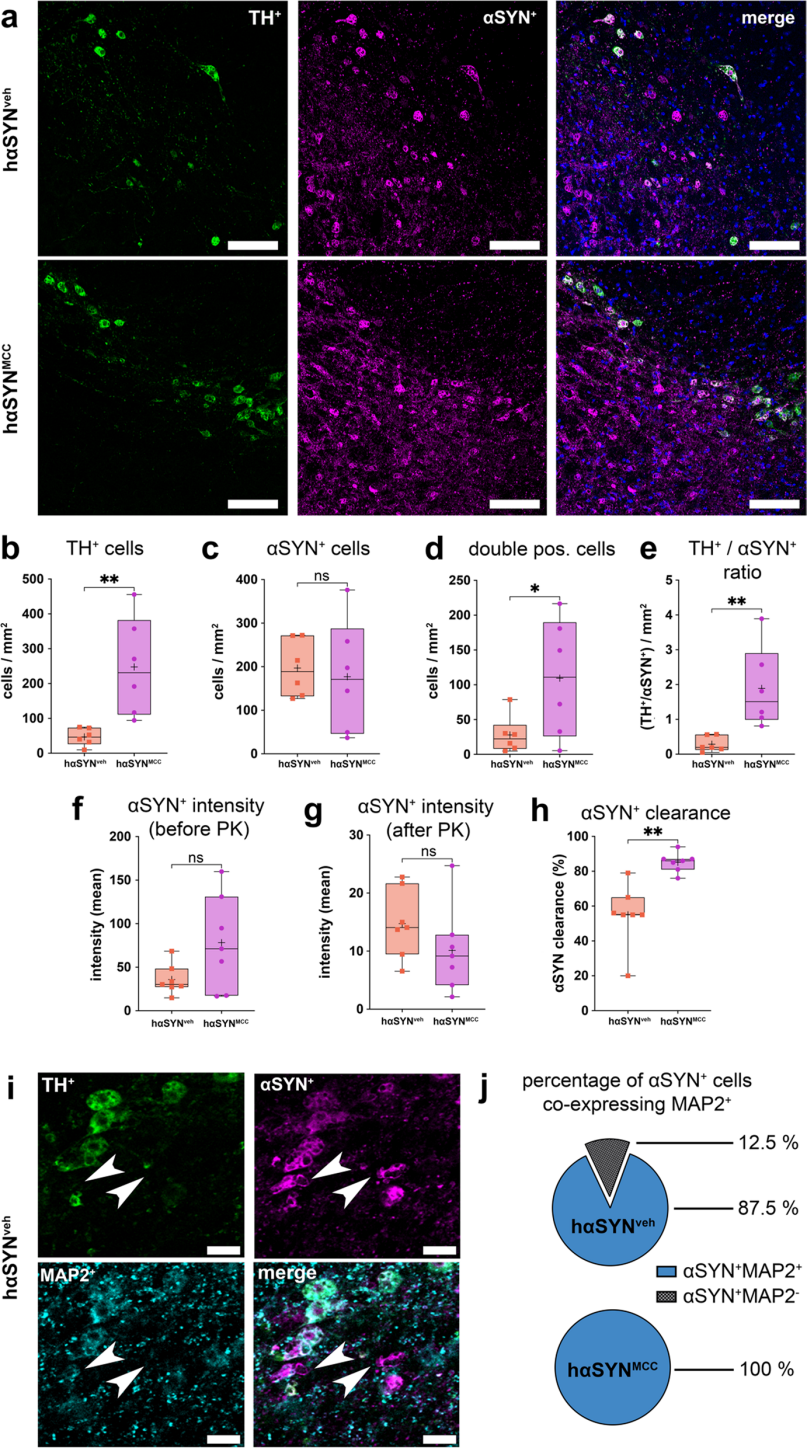
αSYN solubility and distribution analysis in hαSYN mice. **a** Confocal 8-bit images showing TH and αSYN in the substantia nigra (SN) of hαSYN^veh/MCC^ mice. Scale bars 100 µm. **b** TH^+^ cells in hαSYN^MCC^ mice compared to hαSYN^veh^ mice [*t*(10) = 3.432, *P = *0.0064]. **c** Comparison of αSYN^+^ cells in hαSYN^veh/MCC^ mice [*t*(10) = 0.3342, *P = *0.7452, *n* = 6 mice]. **d** TH^+^ and αSYN^+^ double positive cells hαSYN^veh/MCC^ mice. The number of cell per mm^2^ are shown [*t*(10) = 2.239, *P = *0.0491, *n* = 6]. **e** Ratio of TH^+^ /αSYN^+^ cells in hαSYN^veh/MCC^ mice [*t*(10) = 3.307, *P = *0.0079]. **f, g** Mean intensity of αSYN immunoreactivity within the SN before [**f**; *t*(12) = 1.966, *P = *0.0729] or after [**g**; *t*(12) = 1.288, *P = *0.2219] digestion with proteinase K (PK). **h** Relative PK-mediated loss of αSYN immunoreactivity (clearance) within the SN of hαSYN^veh/MCC^ mice [*t*(12) = 4.274, *P = *0.0011]. **i** SN region of vehicle-treated hαSYN mice indicating loss of neuronal somata, demonstrated by loss of anti-MAP2 and anti-TH immunoreactivity, in representative αSYN^+^ clusters (arrowheads, Scale bars 25 µm). **j** Percentage of αSYN^+^ and MAP2^+^ cells in hαSYN^veh/MCC^ mice (Mann–Whitney test two-tailed, *P = *0.0286). Data in **b**–**e** and **j** were accessed from confocal x, y–z image stacks. Data in **f**–**h** were accessed from epifluorescence tile-stack images. Statistics in **b**–**h** result from two-tailed *t* test. *P < 0.05, **P < 0.01, *** *P* < 0.001, ****P < 0.0001

Since the inflammasome is involved in removal of potentially toxic protein aggregates in cells, we investigated the consequences of inflammasome inhibition on p_ath_αSYN aggregation. Therefore, we analyzed the total amount of αSYN^+^ and TH^+^ cells and the solubility of p_ath_αSYN in hαSYN^MCC^/hαSYN^veh^ mice. While TH^+^ cell numbers were significantly decreased in hαSYN^veh^ mice, compared to hαSYN^MCC^ mice (by 81.1%, *P* < 0.01, Fig. 6a, b), we found no differences in the number of αSYN^+^ cells in the SN of hαSYN^MCC^ compared to hαSYN^veh^ mice (Fig. 6a, c). Moreover, significantly fewer αSYN^+^ and TH^+^ double positive cells were found in hαSYN^veh^ mice (by 74.5%, *P* < 0.05, Fig. 6a,d), along with a significantly decreased ratio of TH^+^ to αSYN^+^ cells (by 84.8%, *P* < 0.01, Fig. 6a, e). We then tested whether αSYN immunoreactivity was also accompanied by changes in the solubility of p_ath_αSYN by treating slices with PK to see whether the PK-resistance of p_ath_αSYN would be altered by systemic anti-inflammasome treatment. As indicated by changes in the mean anti-αSYN signal intensity both before (*P* > 0.05, Fig. 6f) and after (*P* > 0.05, Fig. 6g) PK digestion, we observed that relative clearance of p_ath_αSYN was lower in hαSYN^veh^ mice (by 35.4%, *P* < 0.01, Fig. 6h). This suggests a higher amount of soluble p_ath_αSYN in SN neurons of hαSYN^MCC^ mice. Finally, we tested whether there would be more intact αSYN^+^ cells in hαSYN^MCC^ mice. Neurons were co-labeled with anti-αSYN, anti-TH, and the neuron marker MAP2. MAP2 labels were used to outline the somatodendritic area of individual neurons. Surprisingly, we detected a high number of αSYN^+^ cells expressing MAP2 in hαSYN^veh^ mice (mean 87.5%); all (100%) αSYN^+^ cells in hαSYN^MCC^ mice were found to be positive for MAP2 (Fig. 6i, j). The data indicated a reduced aggregation tendency of p_ath_αSYN in hαSYN^MCC^ mice. Furthermore, NLRP3 inhibition also caused an increased viability of DA SN neurons expressing high amounts of p_ath_αSYN.

## Discussion

Strong experimental evidence demonstrates that neuroinflammatory processes contribute to the pathophysiology and progression of PD [3–6, 27, 28]. However, anti-inflammatory treatment has not yet been established as a therapeutic option for PD patients and therapy remains purely symptomatic [2, 29]. Moreover, neuroinflammatory processes are highly heterogeneous, thus raising the question of which element of the multidimensional inflammatory response can and should be efficiently targeted in PD to achieve optimal efficacy. Therefore, we investigated the implications of NLRP3 inhibition in a model of PD. NLRP3 is known to interact with adaptive and innate inflammation and is also highly druggable. Earlier studies investigating properties of NLRP3, studied distinct effects of innate and adaptive inflammation independently, used toxin-related PD models or employed a prophylactic treatment regime [12, 18, 19, 22, 30]. Furthermore, data from toxin-based PD models regarding adaptive immune response should be viewed with caution as contradictory observations have been reported using such models [2, 31]. This and potentially better translatability to human PD were the main reasons that the hαSYN model was employed here.

In the SN of hαSYN^veh^ mice, a model of untreated PD, we found reduced total number of TH^+^ cells but increased total NLRP3 expression, specifically in CD11b^+^ microglia, and increased CD4^+^ and CD8^+^ T cells compared to the EV^veh^ control condition. Increased amounts of insoluble p_ath_αSYN could also be found in this pro-inflammatory environment of modelling PD. In hαSYN^MCC^ mice, a model of treating PD with an NLRP3 inhibitor, we observed increased DA cell survival. These hαSYN^MCC^ mice also displayed a significant decrease in the innate and adaptive immune responses, along with a significantly reduced amount of insoluble, aggregated p_ath_αSYN. Our results indicate that the innate and adaptive immune response are likely interwoven in PD progression and p_ath_αSYN aggregation. Accordingly, anti-inflammatory treatment in p_ath_αSYN-related PD may provide scope for cellular survival processes and sufficiently halt disease progression. It is important to state that the model we used is a progressive disease model and treatment was initiated after model generation—not before, as in an earlier study of a p_ath_αSYN-related PD model [19]. Therefore, our results demonstrate that an ongoing pathology can be sufficiently modified by continuous systemic inflammasome inhibition of 20 mg/kg i.p. every other day. Dosages of 20 mg/kg (oral) are known to reduce NLRP3 activation significantly and cross the blood–brain-barrier [13, 19].

There are three main implications of our research. First, as PD is a progressive disease driven by spreading of p_ath_αSYN and DA neurodegeneration, we have shown that targeted inflammasome inhibition might be a therapeutic option for PD treatment—even though pharmaceutical inflammasome inhibition was initiated early in the disease process. Second, combined with the results from our recent study in the same animal model [6], the role of the adaptive immune response in p_ath_αSYN aggregation and subsequent neurodegeneration should not be underestimated. Further studies to investigate the distinct effects and interactions of T cell immunity, p_ath_αSYN aggregation, and neurodegeneration are needed. In particular, the role of p_ath_αSYN as a pathological antigen in the origin of altered cellular survival mechanisms is not yet fully understood. In line with this observation, MCC950 was shown to reduce hippocampal p_ath_αSYN accumulation in a mouse model for dementia with Lewy bodies by mTOR-mediated autophagy degradation [32]. Since antigen recognition triggers mTOR activation in T cells [33], this underlines the importance that T cell targeting treatments might lead to sufficient treatment of human PD. Third, we observed a surprisingly high amount of MAP2^+^ cells in hαSYN^veh^ mice but with significant loss of TH^+^ signal; this shows the importance of also investigating treatment options at a later stage of the disease. However, this was not addressed in our study.

In summary, NLRP3 inhibition led to a profound decrease in innate and adaptive neuroinflammation and reduced p_ath_αSYN aggregation, resulting in improved cellular coping mechanisms and DA cell rescue. This was accompanied by non-significant changes in the absolute p_ath_αSYN load but with an increased amount of soluble p_ath_αSYN in inflammasome-inhibited PD animals. In support of this finding, unaltered total amounts of αSYN after MCC950 treatment in a p_ath_αSYN-related PD model has been described earlier [19].

One major limitation of our study is that we did not show how inflammasome inhibition contributed to better intracellular ‘acceptance’ of the hαSYN protein, and whether this treatment reduced the antigen-like, inflammatory properties of p_ath_αSYN (A53T). Furthermore, the consequences of pausing or stopping inflammasome inhibition are unknown; we found a high number of TH^+^ cells expressing increased amounts p_ath_αSYN (in hαSYN^MCC^ mice) that might be able to further aggregate in case of medication discontinuation. This would be of specific interest for ethical considerations in future clinical studies.

## Conclusion

The inflammasome inhibitor MCC950 protected DA neurons of the SN from pathological α-synuclein-mediated neurodegeneration and attenuated pathological α-synuclein-related neuroinflammation in a PD mouse model. This suggests that targeting cellular immunity might be an efficient mechanism-based strategy for reducing the impact of α-synuclein pathology in PD.

## Data Availability

The data sets used and/or analyzed during the current study are available from the corresponding author on reasonable request.

## Abbreviations

A53T-αSYN: Human A53T-α-synuclein
BSA: Bovine serum albumin
EV: Empty vector
hαSYN: AAV1/2-A53T-α-synuclein
MPTP: 1‑Methyl‑4‑phenyl‑1,2,3,6‑tetrahydropyridine
NGS: Normal goat serum
NLRP3: NOD-, LRR- and pyrin domain-containing 3
OD: Optical density
p_ath_αSYN: Pathological a-synuclein
PBS: Phosphate-buffered saline
PBT: Phosphate-buffered saline 0.1 M with 0.5% Triton-X 100
PD: Parkinson’s disease
PFA: Paraformaldehyde
PK: Proteinase K
ROI: Region of interest
RT: Room temperature
SNpc: SN pars compacta
SEM: Standard error of the mean
SN: Substantia nigra
TH: Tyrosine hydroxylase
